# Supplementary material for: Antigene MYCN Silencing by BGA002 Inhibits SCLC Progression Blocking mTOR Pathway and Overcomes Multidrug Resistance
Source: Cancers (Basel). 2023 Feb 3;15(3):990. doi: 10.3390/cancers15030990 (PMC9913109; doi:10.3390/cancers15030990)
Supplement: Supplementary file 1 [file cancers-15-00990-s001.zip › Figures S1-S7, Tables S1-S7.docx]

Article

Antigene *MYCN* Silencing by BGA002 Inhibits SCLC
Progression Blocking mTOR Pathway and Overcomes
Multidrug Resistance

Sonia Bortolotti ^1,†^, Silvia Angelucci ^1,†^, Luca Montemurro ^2^, Damiano Bartolucci ^1^, Salvatore Raieli ^3^,
Silvia Lampis ^4^, Camilla Amadesi ^1^, Annalisa Scardovi ^1^, Giammario Nieddu ^1^, Lucia Cerisoli ^1^,
Francesca Paganelli ^5,6^, Francesca Chiarini ^7^, Gabriella Teti ^5^, Mirella Falconi ^5^, Andrea Pession ^8^, Patrizia Hrelia ^9^ and Roberto Tonelli ^9,^*

^1^ R&D Department, BIOGENERA SpA, 40064 Bologna, Italy; sonia.bortolotti@biogenera.com (S.B.); silvia.angelucci@biogenera.com (S.A.); damiano.bartolucci@biogenera.com (D.B.); camilla.amadesi@biogenera.com (C.A.); annalisa.scardovi@biogenera.com (A.S.); giammario.nieddu@biogenera.com (G.N.); lucia.cerisoli@biogenera.com (L.C.)

^2^ Pediatric Oncology and Hematology Unit, IRCCS, Azienda Ospedaliero-Universitaria di Bologna,
40138 Bologna, Italy; lucam.ageop@aosp.bo.it

^3^ Oncodesign SA, 21079 Dijon, France; salvatore.raieli2@gmail.com

^4^ Research Laboratories-Oncohematology Department, Bambino Gesu Children’s Hospital, IRCCS,
00165 Rome, Italy; silvia.lampis@opbg.net

| **Citation:** Bortolotti, S.; Angelucci, S.; Montemurro, L.; Bartolucci, D.; Raieli, S.; Lampis, S.; Amadesi, C.; Scardovi, A.; Nieddu, G.; Cerisoli, L.; et al. Antigene *MYCN* Silencing by BGA002 Inhibits SCLC Progression Blocking mTOR Pathway and Overcomes Multidrug Resistance. *Cancers* **2023**, *15*, x. https://doi.org/10.3390/xxxxx  Academic Editor(s):  Received: 10 January 2023  Revised: 27 January 2023  Accepted: 30 January 2023  Published: date  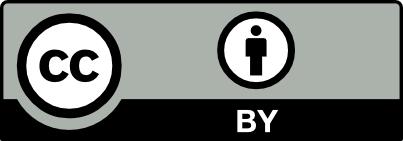  **Copyright:** © 2023 by the authors. Submitted for possible open access publication under the terms and conditions of the Creative Commons Attribution (CC BY) license (https://creativecommons.org/licenses/by/4.0/). |
| --- |

^5^ Alma Mater Studiorum, Department of Biomedical and Neuromotor Sciences, University of Bologna,
40126 Bologna, Italy; francesca.paganell16@unibo.it (F.P.); gabriella.teti2@unibo.it (G.T.);
mirella.falconi@unibo.it (M.F.)

^6^ Unit of Bologna, CNR Institute of Molecular Genetics “Luigi Luca Cavalli-Sforza”, 40129 Bologna, Italy

^7^ Department of Bio-medical, Metabolic and Neural Sciences, Section of Human Morphology,
University of Modena and Reggio Emilia, 41125 Modena, Italy; francesca.chiarini@unimore.it

^8^ Pediatric Unit, IRCCS, Azienda Ospedaliero-Universitaria di Bologna, 40138 Bologna, Italy;
andre-a.pession@unibo.it

^9^ Department of Pharmacy and Biotechnology, University of Bologna, 40126 Bologna, Italy;
patrizia.hrelia@unibo.it

***** Correspondence: roberto.tonelli@unibo.it

† These authors contributed equally to this work.

**Figure S1.** SCLC cell lines response to BGA002 treatment. (**A**) MYCN mRNA basal expression is reported for each SCLC cell line related to unexpressing cell line. (**B**) EC_50_ is reported for both mRNA expression and viability for each SCLC cell line. (**C**) BGA002 dose response curve for both mRNA and viability for each SCLC cell line.


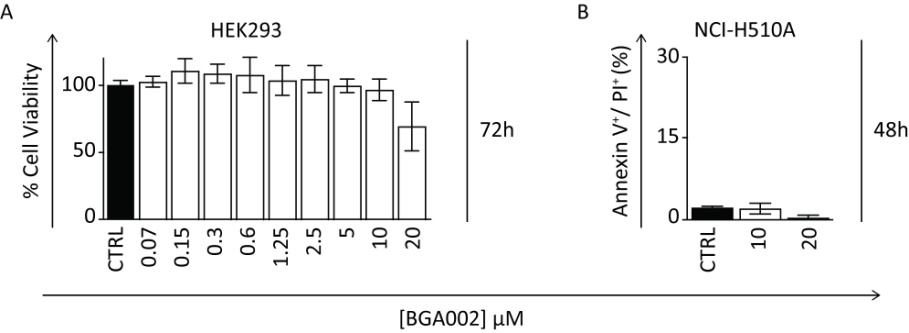


**Figure S2.** BGA002 does not reduce cell viability and apoptosis activation in the absence of *MYCN* expression. (A) Cell viability evaluation in non-tumorigenic cell line with *MYCN* unexpression, after treatment with BGA002 at different doses. (B) Apoptosis percentage in *MYCN*-unexpressing SCLC cell line after treatment with BGA002.


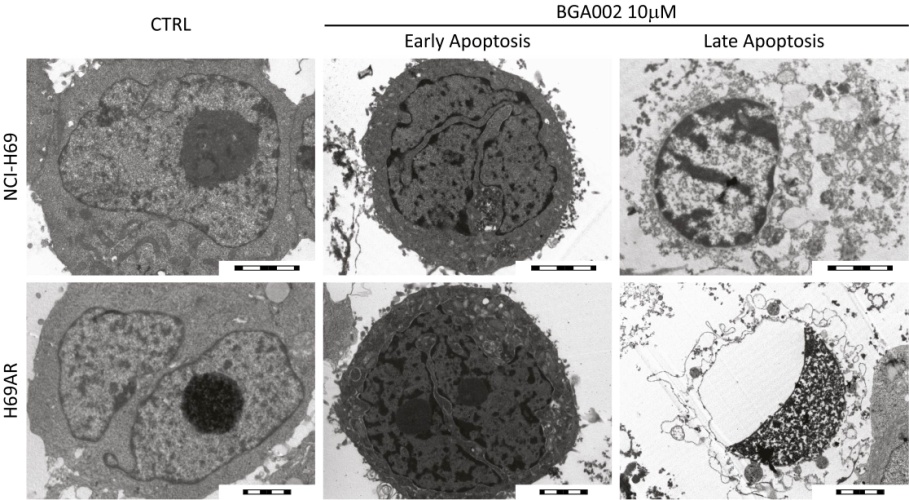


**Figure S3.** SCLC cell lines treated with BGA002 show apoptosis signature structures. Electron microscopy images of NCI-H69 and H69AR before and after treatment with BGA002 10uM for 48 hours. Signatures structures appear for both early and late apoptosis.


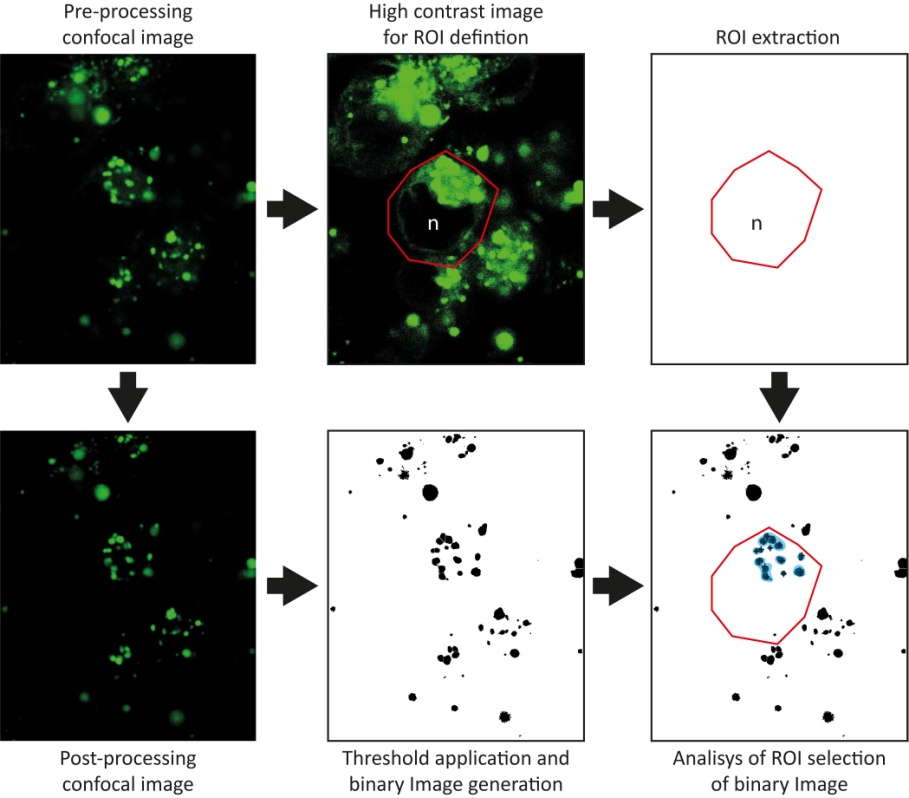


**Figure S4.** Schematic representation of the lysosomes analysis approach. As reported, images are contrasted to define region of interest in correspondence of cells, or binarized to measure lysosome structures. Intersection of this two is further analyzed to obtain lysosomes distribution in each cell. Analysis was performed using Fiji package of ImageJ.


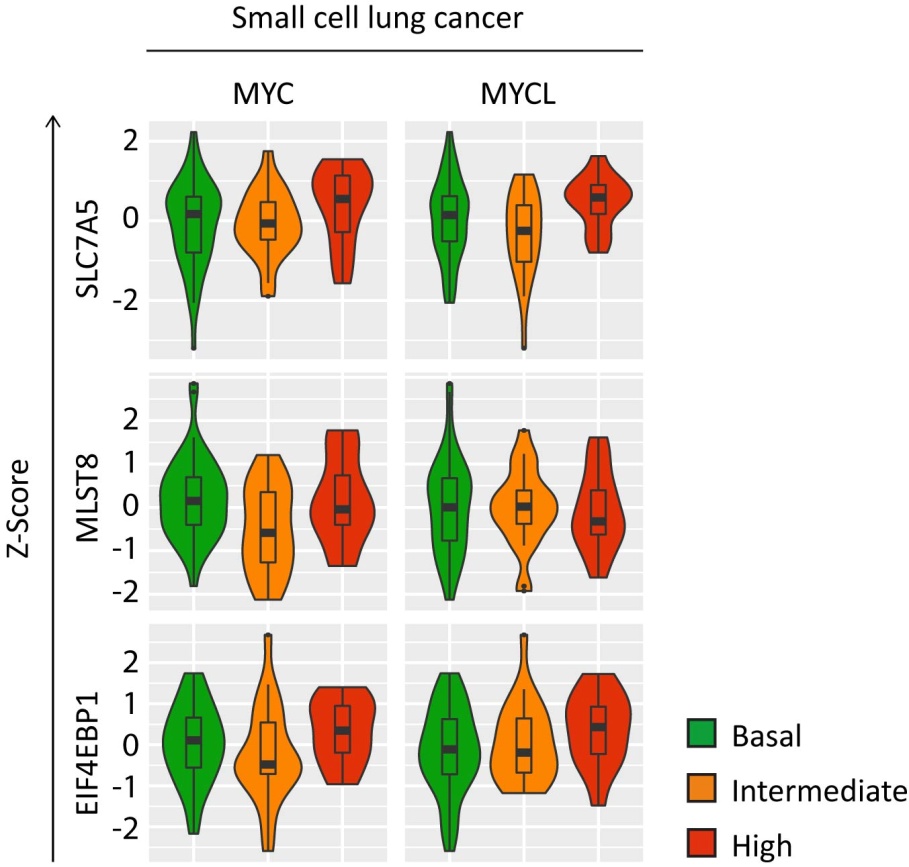


**Figure S5.** mTOR related genes are differentially expressed compared to *MYC* or *MYCL* expression in SCLC patients. mRNA expression of *SLC7A5*, *MLST8* and *EIF4EBP1* in a small cell lung cancer dataset (EGAS00001000925). The violin plots represent normalized (z-score) mRNA expression *MYC* or *MYCL* low to high expressed patients. Each dot represents an individual sample; the middle line indicates the median.


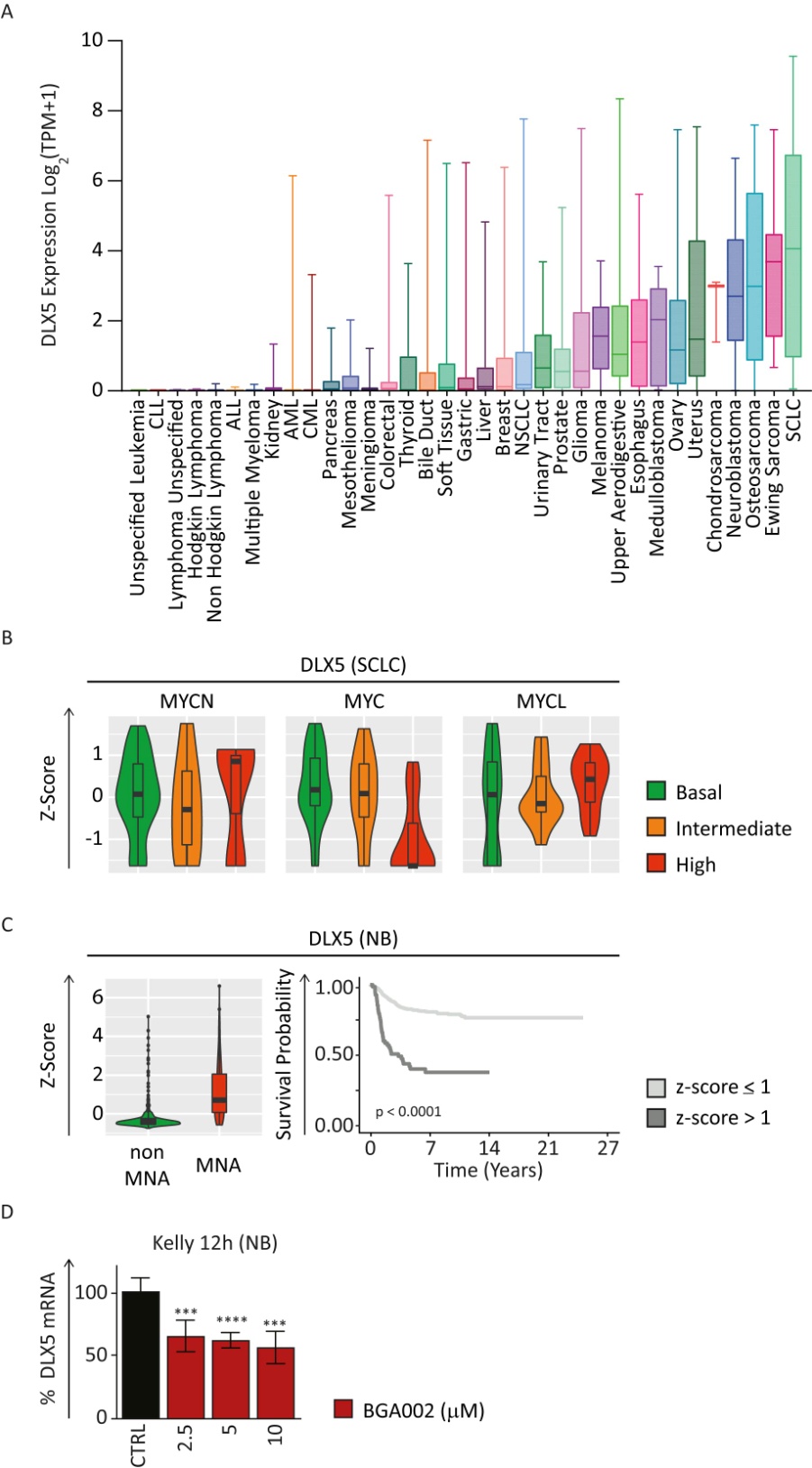


**Figure S6.** *DLX5* is upregulated in SCLC and is downregulated by BGA002. (**A**) Box-plot of CCLE expression data of *DLX5* in overall human tumor cell lines, the middle line indicates the mean. (**B**) mRNA expression of *DLX5* in a small cell lung cancer dataset (EGAS00001000925). The violin plots represent normalized (z-score) mRNA expression for *MYCN*, *MYC* or *MYCL* low to high expressed patients. (**C**) mRNA expression of *DLX5* in a neuroblastoma dataset (E-MTAB-1781). The violin plot represents normalized (z-score) mRNA expression for non-MNA and MNA patients. Each dot represents an individual sample; the middle line indicates the median. Kaplan–Meier plots for the probability of overall survival over time for neuroblastoma patients (E-MTAB-1781). The dark gray line indicates patients with normalized gene expressions higher than 1 (z-score > 1). The p-value is indicated in the middle (Log-rank test). (**D**) BGA002 reduces *DLX5* expression in MNA-NB cells at different doses after 12 hours of treatment.


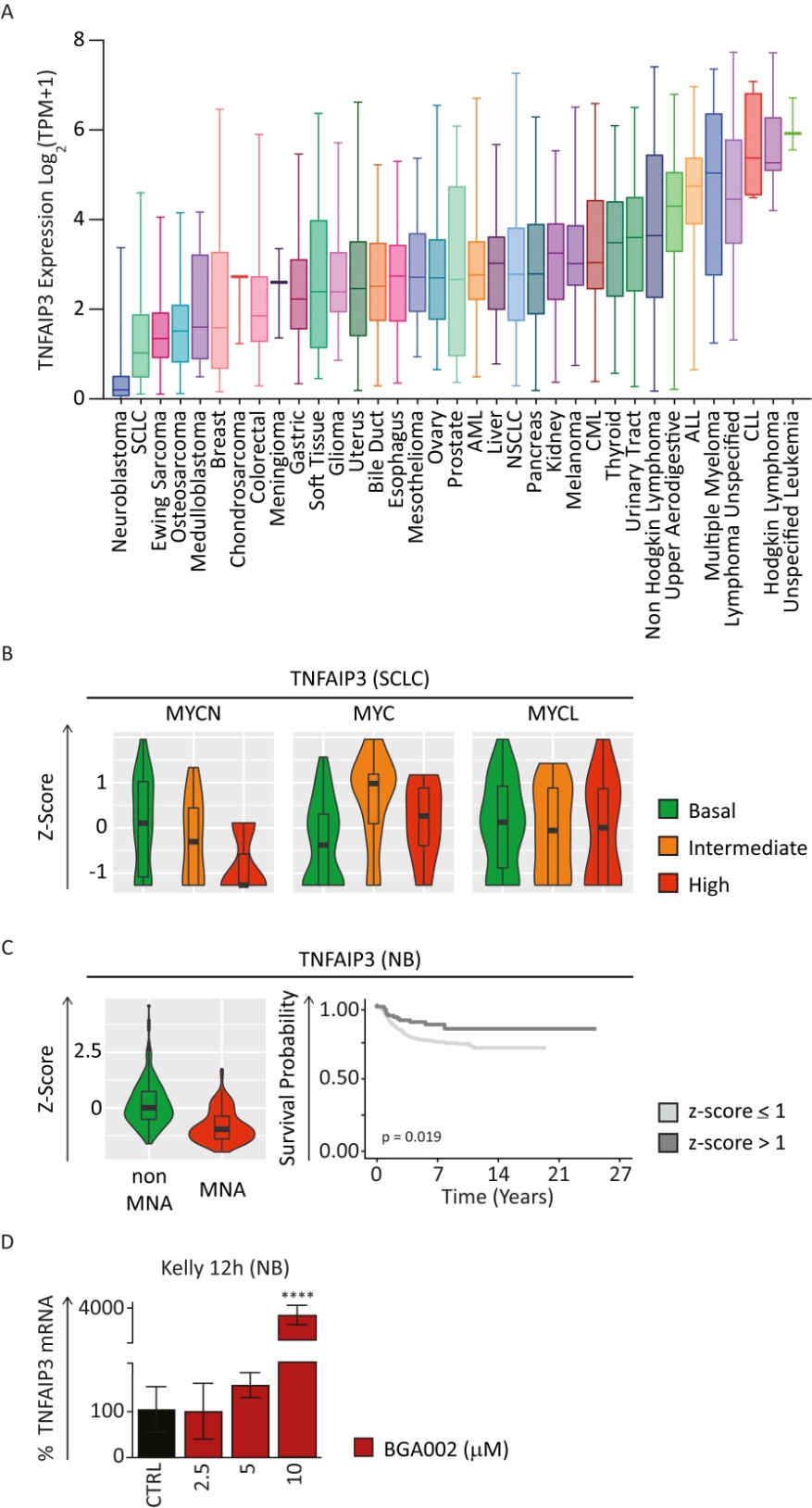


**Figure S7.** *TNFAIP3* is downregulated in SCLC and is upregulated by BGA002. (**A**) Box-plot of CCLE expression data of *TNFAIP3* in overall human tumor cell lines, the middle line indicates the mean. (**B**) mRNA expression of *TNFAIP3* in a small cell lung cancer dataset (EGAS00001000925). The violin plots represent normalized (z-score) mRNA expression for *MYCN*, *MYC* or *MYCL* low to high expressed patients. (**C**) mRNA expression of *TNAFAIP3* in a neuroblastoma dataset (E-MTAB-1781). The violin plot represents normalized (z-score) mRNA expression for non-MNA and MNA patients. Each dot represents an individual sample; the middle line indicates the median. Kaplan–Meier plots for the probability of overall survival over time for neuroblastoma patients (E-MTAB-1781). The dark gray line indicates patients with normalized gene expressions higher than 1 (z-score > 1). The p-value is indicated in the middle (Log-rank test). (**D**) BGA002 reduces *TNFAIP3* expression in neuroblastoma cell lines at different doses after 12 hours of treatment.

**Table S1.** List of cell line used in this study. Table reports for each cell lines, source, mycoplasma status, *MYCN* status and site from who cells are derived. Phase of the therapy and age of the patients are also reported. Citations are listed.

| Cell Line | Site | Age of Patients | Phase of Therapy | MYCN Amplification | Source | Mycoplasma | Citations |
| --- | --- | --- | --- | --- | --- | --- | --- |
| NCI-H69 | Pleural effusion | 55 years | Post-therapy | MYCN amplification | ATCC | Neg | ATCC Cat# HTB-119, RRID:CVCL_1579 |
| H69AR | Lung | 55 years | Post-therapy | MYCN amplification | ATCC | Neg | ATCC Cat# CRL-11351, RRID:CVCL_3513 |
| NCI-N592 | Bone marrow | 55 years | Post-therapy | MYCN amplification | Kindly gifted by Dr. Silvano Ferrini | Neg | RRID:CVCL_A590 |
| NCI-H526 | Bone marrow | 55 years | No prior treatment | MYCN amplification | ATCC | Neg | ATCC Cat# CRL-5811, RRID:CVCL_1569 |
| GLC-14 | Supraclavicular lymph node | 55 years | No prior treatment | MYCN amplification | Kindly gifted by Prof. Elisabeth G E de Vries | Neg | RRID:CVCL_8204, RRID:CVCL_8204 |
| DMS 79 | Pleural effusion | 65 years | Post-therapy | MYCN not amplified-MYCN overexpression | ATCC | Neg | ATCC Cat# CRL-2049, RRID:CVCL_1178 |
| NCI-H510A | Adrenal metastasis | 56 years | Post-therapy | MYCN not expressing | ATCC | Neg | ATCC Cat# HB-184, RRID:CVCL_1565 |
| HEK-293 | Kidney | n/a | n/a | MYCN not expressing | DMSZ | Neg | DSMZ Cat# ACC 305, RRID:CVCL_0045 |

**Table S2.** List of primers used in this study. Gene name for each mRNA analyzed is reported. Forward and reverse sequences are associated are provided both.

| Primer | Sense | Antisense |
| --- | --- | --- |
| MYCN I | ACCCGGAGACACCCGCGCAGAATC | GTAGAAGCAGGGCTGTAGCGAGTC |
| BIRC4 | ACAAGGAGCAGCTTGCAAGA | AGCATGTTGTTCCCAAGGGT |
| SLC7A5 | CTCTTCCTGATCGCCGTCTC | GACCACCTGCATGAGCTTCT |
| SLC1A5 | ACCATATCTCCTTGATCCTGGC | TACGGTCCACGTAATTTTGGAG |
| AKT1 | GCACAAACGAGGGGAGTACA | AAGGTGCGTTCGATGACAGT |
| AKT1S1 | TGAGCCCACAGAGACAGAGA | CGGGGTCTGACTCACAGAAG |
| AKT2 | TGATGGAGTATGCCAACGGG | GTCCTCCAGCACCTTGATGT |
| MLST8 | TGGCAGCTGTCAATAGCACC | TCTTGATGCTCAGCTCCGTC |
| RPTOR | GGTGCTGTTAAGCCAAGTGC | TAGGGGAAGATGCCGACAGA |
| EIF4EBP1 | ACCTGTGACCAAAACACCCC | GGTAGTGCTCCACACGATGG |
| ACACA | CAGAGGGAACATCCCTACGC | AAGAGACCATTCCGCCCATC |
| FASN | AGCAGTTCACGGACATGGAG | ATGGTACTTGGCCTTGGGTG |
| ATXN2 | GGAACGTGGTCATCAGTGGT | CAGCTTGGGGAGAAGCAAGA |
| ATXN2L | CAGCCATTGCCATGAACTCG | GCTCTCGCTGACGAAACTCT |
| SAM4DB | CCCCAGGCCATTCTCATGTT | GTCTGTCCCATCACCCAAGG |
| EIF4G1 | ACCTGTGTGACGAGCAGAAG | ACGGAGCCACTTGAAGAAGG |
| NBAS | CAGTACCGAAGACACTGGGATT | CATTCCCCTTGTTGCTTCAGAG |
| NANOS1 | TGTTGGGGGTCCTTCATGTG | TGAGGGTGGGAGGGTAAAGT |
| RELA | TGTATTTCACGGGACCAGGC | GGTCCGCTGAAAGGACTCTT |
| NFKB1 | ATCTGCACTGTAACTGCTGGA | TGGCGGATTAGCTCTTTTTCC |
| TP73 | TCCGCGTGGAAGGCAATAAT | ATGGTGGTGAATTCCGTCCC |
| TNFAIP3 | ATCAAAATGGCTTCCACAGAC | TGGAGAGGCAAGTAAATTCCAC |
| CRTC2 | GAGGGACGGGGAAGGAAGAT | TGTGTATGCCAGTCGCAGTT |
| MDH2 | GTTCAACACCAATGCCACGATT | AAACTTCTGCTGTGATGGGGATG |
| G6PC3 | GGGTCCATGAGTCTGGTTACTAC | CTGGTGAGGGAAATGTGCTAAGAT |
| IGF2 | TCGTTGAGGAGTGCTGTTTC | GTATCTGGGGAAGTTGTCCG |
| WDTC1 | GGACATGGGCTCCTGAAGTT | GGGGTCAGTGACATGGTAGC |
| G6PD | CCTTCCATCAGTCGGATACACA | ATAGCCCACGATGAAGGTGTTT |

**Table S3.** Lysosomes raw data and statistical analysis. Lysosomes count is reported from 0 to 5 μm each 0,1μm. Paired T-test is reported considering 0-2μm diameters range. For analysis a total of n = 40 cells were used.

| Diameter_Bin_Intervall | NCI-H69_CTRL | NCI-H69_BGA002 | H69AR_CTRL | H69AR_BGA002 | NCI-N592_CTRL | NCI-N592_BGA002 |
| --- | --- | --- | --- | --- | --- | --- |
| Diameter_0-0,1_(μm) | 0 | 0 | 0 | 0 | 0 | 0 |
| Diameter_0,1-0,2_(μm) | 0 | 0 | 0 | 0 | 0 | 0 |
| Diameter_0,2-0,3_(μm) | 0 | 0 | 0 | 0 | 0 | 0 |
| Diameter_0,3-0,4_(μm) | 16 | 17 | 8 | 17 | 4 | 19 |
| Diameter_0,4-0,5_(μm) | 41 | 40 | 10 | 42 | 4 | 18 |
| Diameter_0,5-0,6_(μm) | 31 | 29 | 9 | 23 | 6 | 7 |
| Diameter_0,6-0,7_(μm) | 26 | 36 | 10 | 17 | 2 | 12 |
| Diameter_0,7-0,8_(μm) | 22 | 34 | 10 | 16 | 1 | 5 |
| Diameter_0,8-0,9_(μm) | 13 | 20 | 1 | 18 | 0 | 2 |
| Diameter_0,9-1_(μm) | 10 | 19 | 3 | 13 | 0 | 0 |
| Diameter_1-1,1_(μm) | 9 | 14 | 3 | 24 | 0 | 2 |
| Diameter_1,1-1,2_(μm) | 6 | 17 | 1 | 10 | 0 | 0 |
| Diameter_1,2-1,3_(μm) | 3 | 12 | 0 | 12 | 0 | 0 |
| Diameter_1,3-1,4_(μm) | 4 | 8 | 0 | 10 | 0 | 0 |
| Diameter_1,4-1,5_(μm) | 6 | 6 | 0 | 4 | 0 | 0 |
| Diameter_1,5-1,6_(μm) | 2 | 4 | 0 | 3 | 0 | 1 |
| Diameter_1,6-1,7_(μm) | 3 | 1 | 0 | 2 | 0 | 0 |
| Diameter_1,7-1,8_(μm) | 0 | 1 | 0 | 2 | 0 | 1 |
| Diameter_1,8-1,9_(μm) | 0 | 0 | 0 | 1 | 0 | 0 |
| Diameter_1,9-2_(μm) | 0 | 0 | 0 | 0 | 0 | 0 |
| Diameter_2-2,1_(μm) | 0 | 0 | 0 | 1 | 0 | 0 |
| Diameter_2,1-2,2_(μm) | 1 | 0 | 0 | 0 | 0 | 0 |
| Diameter_2,2-2,3_(μm) | 0 | 0 | 0 | 0 | 0 | 0 |
| Diameter_2,3-2,4_(μm) | 0 | 0 | 0 | 0 | 0 | 0 |
| Diameter_2,4-2,5_(μm) | 0 | 0 | 0 | 0 | 0 | 0 |
| Diameter_2,5-2,6_(μm) | 0 | 0 | 0 | 0 | 0 | 0 |
| Diameter_2,6-2,7_(μm) | 0 | 0 | 0 | 0 | 0 | 0 |
| Diameter_2,7-2,8_(μm) | 0 | 0 | 0 | 0 | 0 | 0 |
| Diameter_2,8-2,9_(μm) | 0 | 0 | 0 | 0 | 0 | 0 |
| Diameter_2,9-3_(μm) | 0 | 0 | 0 | 0 | 0 | 0 |
| Diameter_3-3,1_(μm) | 0 | 0 | 0 | 0 | 0 | 0 |
| Diameter_3,1-3,2_(μm) | 0 | 0 | 0 | 0 | 0 | 0 |
| Diameter_3,2-3,3_(μm) | 0 | 0 | 0 | 0 | 0 | 0 |
| Diameter_3,3-3,4_(μm) | 0 | 0 | 0 | 0 | 0 | 0 |
| Diameter_3,4-3,5_(μm) | 0 | 0 | 0 | 0 | 0 | 0 |
| Diameter_3,5-3,6_(μm) | 0 | 0 | 0 | 0 | 0 | 0 |
| Diameter_3,6-3,7_(μm) | 0 | 0 | 0 | 0 | 0 | 0 |
| Diameter_3,7-3,8_(μm) | 0 | 0 | 0 | 0 | 0 | 0 |
| Diameter_3,8-3,9_(μm) | 0 | 0 | 0 | 0 | 0 | 0 |
| Diameter_3,9-4_(μm) | 0 | 0 | 0 | 0 | 0 | 0 |
| Diameter_4-4,1_(μm) | 0 | 0 | 0 | 0 | 0 | 0 |
| Diameter_4,1-4,2_(μm) | 0 | 0 | 0 | 0 | 0 | 0 |
| Diameter_4,2-4,3_(μm) | 0 | 0 | 0 | 0 | 0 | 0 |
| Diameter_4,3-4,4_(μm) | 0 | 0 | 0 | 0 | 0 | 0 |
| Diameter_4,4-4,5_(μm) | 0 | 0 | 0 | 0 | 0 | 0 |
| Diameter_4,5-4,6_(μm) | 0 | 0 | 0 | 0 | 0 | 0 |
| Diameter_4,6-4,7_(μm) | 0 | 0 | 0 | 0 | 0 | 0 |
| Diameter_4,7-4,8_(μm) | 0 | 0 | 0 | 0 | 0 | 0 |
| Diameter_4,8-4,9_(μm) | 0 | 0 | 0 | 0 | 0 | 0 |
| Diameter_4,9-5_(μm) | 0 | 0 | 0 | 0 | 0 | 0 |
| Diameter_5-5,1_(μm) | 0 | 0 | 0 | 0 | 0 | 0 |
| Diameter_5,1-5,2_(μm) | 0 | 0 | 0 | 0 | 0 | 0 |
| Diameter_5,2-5,3_(μm) | 0 | 0 | 0 | 0 | 0 | 0 |
| Diameter_5,3-5,4_(μm) | 0 | 0 | 0 | 0 | 0 | 0 |
| Diameter_5,4-5,5_(μm) | 0 | 0 | 0 | 0 | 0 | 0 |
| Paired Student's t-test (Diameter_0-2μm) |  | 0.005146413 |  | 0.000404192 |  | 0.028555059 |

**Table S4.** Weight assessment in SCLC mouse model. Table reports information about animals used in this study. For each animal we provide, animal unique code, treatment administered, pre-on study weight, weight at the end of the study and weight relative variation. Data refers to SCLC mouse model (NCI-H69-Luc).

| Animal_Ref._Number | Treatment | Pre_On_Study_Weight | End_Study_Weight | Relative_Body_Weight_Variation |
| --- | --- | --- | --- | --- |
| 1461 | NaCl_0.9% | 18.9 | 22.1 | + 0.169 |
| 1465 | NaCl_0.9% | 16.8 | 18.2 | + 0.083 |
| 1473 | NaCl_0.9% | 13.5 | 18 | + 0.333 |
| 1475 | NaCl_0.9% | 19.6 | 22.9 | + 0.168 |
| 1483 | NaCl_0.9% | 17.6 | 21.2 | + 0.205 |
| 1489 | NaCl_0.9% | 16.5 | 20.3 | + 0.23 |
| 1447 | NaCl_0.9% | 17.1 | 21.9 | + 0.281 |
| 1448 | NaCl_0.9% | 18.7 | 24.2 | + 0.294 |
| 1449 | NaCl_0.9% | 19.4 | 22.7 | + 0.17 |
| 1450 | NaCl_0.9% | 20.1 | 24.5 | + 0.219 |
| 1451 | NaCl_0.9% | 17.8 | 20.4 | + 0.146 |
| 1452 | NaCl_0.9% | 19.5 | 24.2 | + 0.241 |
| 1464 | BGA002_50mg/kg/day | 16.1 | 18.9 | + 0.174 |
| 1467 | BGA002_50mg/kg/day | 15.8 | 19.7 | + 0.247 |
| 1471 | BGA002_50mg/kg/day | 21.3 | 23.9 | + 0.122 |
| 1482 | BGA002_50mg/kg/day | 17.2 | 22.3 | + 0.297 |
| 1485 | BGA002_50mg/kg/day | 18 | 22.4 | + 0.244 |
| 1488 | BGA002_50mg/kg/day | 17.5 | 22.5 | + 0.286 |
| 1491 | BGA002_50mg/kg/day | 15.7 | 18.5 | + 0.178 |
| 1493 | BGA002_50mg/kg/day | 15.8 | 22.4 | + 0.418 |

**Table S5.** Weight assessment in multidrug-resistant SCLC mouse model. Table reports information about animals used in this study. For each animal we provide, animal unique code, treatment administered, pre-on study weight, weight at the end of the study and weight relative variation. Data refers to multidrug-resistant SCLC mouse model (H69AR-Luc).

| Animal_Ref._Number | | Treatment | Pre_On_Study_Weight | End_Study_Weight | Relative_Body_Weight_Variation |
| --- | --- | --- | --- | --- | --- |
| 1132 | NaCl_0.9% | | 18.1 | 25.2 | + 0.39 |
| 1135 | NaCl_0.9% | | 24.2 | 31.4 | + 0.3 |
| 1138 | NaCl_0.9% | | 23.1 | 30 | + 0.3 |
| 1140 | NaCl_0.9% | | 17.3 | 20.9 | + 0.21 |
| 1141 | NaCl_0.9% | | 18.4 | 23.7 | + 0.29 |
| 1145 | NaCl_0.9% | | 21.9 | 25.5 | + 0.16 |
| 1148 | NaCl_0.9% | | 21.1 | 30.7 | + 0.45 |
| 1150 | NaCl_0.9% | | 19.8 | 27 | + 0.36 |
| 1151 | NaCl_0.9% | | 18.2 | 25.2 | + 0.38 |
| 1153 | NaCl_0.9% | | 20.1 | 25.3 | + 0.26 |
| 1156 | NaCl_0.9% | | 20.1 | 27.2 | + 0.35 |
| 1163 | NaCl_0.9% | | 16.5 | 23 | + 0.39 |
| 1183 | NaCl_0.9% | | 20.4 | 26 | + 0.27 |
| 1184 | NaCl_0.9% | | 19.9 | 24.5 | + 0.23 |
| 1191 | NaCl_0.9% | | 20.1 | 26.6 | + 0.32 |
| 1192 | NaCl_0.9% | | 20.7 | 25.6 | + 0.24 |
| 1128 | BGA002_50mg/kg/day | | 15.5 | 21.7 | + 0.4 |
| 1133 | BGA002_50mg/kg/day | | 17.8 | 23.5 | + 0.32 |
| 1136 | BGA002_50mg/kg/day | | 22.2 | 30.1 | + 0.36 |
| 1142 | BGA002_50mg/kg/day | | 19.6 | 24.5 | + 0.25 |
| 1146 | BGA002_50mg/kg/day | | 22.8 | 27.7 | + 0.21 |
| 1147 | BGA002_50mg/kg/day | | 21.6 | 30.8 | + 0.43 |
| 1149 | BGA002_50mg/kg/day | | 18.7 | 27.8 | + 0.49 |
| 1152 | BGA002_50mg/kg/day | | 16.6 | 24.5 | + 0.48 |
| 1154 | BGA002_50mg/kg/day | | 19.5 | 27.2 | + 0.39 |
| 1155 | BGA002_50mg/kg/day | | 20.1 | 29.5 | + 0.47 |
| 1157 | BGA002_50mg/kg/day | | 21.1 | 26 | + 0.23 |
| 1158 | BGA002_50mg/kg/day | | 20.8 | 26.5 | + 0.27 |
| 1159 | BGA002_50mg/kg/day | | 23.7 | 34.6 | + 0.46 |
| 1185 | BGA002_100mg/kg/day | | 22.1 | 28.6 | + 0.29 |
| 1187 | BGA002_100mg/kg/day | | 19.6 | 26.1 | + 0.33 |
| 1189 | BGA002_100mg/kg/day | | 20.3 | 27.2 | + 0.34 |
| 1190 | BGA002_100mg/kg/day | | 20.4 | 25.5 | + 0.25 |
| 1193 | BGA002_100mg/kg/day | | 23.8 | 30 | + 0.26 |
| 1194 | BGA002_100mg/kg/day | | 18 | 23.4 | + 0.3 |

**Table S6.** MYCN mRNA EC_50_ (μM). Table reports EC_50_ values calculated for each experiment conducted on cell lines used in the study, after BGA002 treatment, using RT-qPCR.

| **Cell Lines** | **MNA** | **Non-MNA** |
| --- | --- | --- |
| NCI-H69 | 0.29 |  |
| NCI-H69 | 0.28 |  |
| NCI-H69 | 0.11 |  |
| NCI-H69 | 0.36 |  |
| H69AR | 0.17 |  |
| H69AR | 0.35 |  |
| H69AR | 0.71 |  |
| NCI-N592 | 0.35 |  |
| NCI-N592 | 0.21 |  |
| NCI-N592 | 0.18 |  |
| NCI-H526 | 0.88 |  |
| NCI-H526 | 0.47 |  |
| NCI-H526 | 1.75 |  |
| GLC-14 | 1.47 |  |
| GLC-14 | 1.83 |  |
| GLC-14 | 0.06 |  |
| DMS 79 |  | 0.45 |
| DMS 79 |  | 0.54 |
| DMS 79 |  | 1.25 |
| Mean | 0.59 | 0.75 |
| Test-T | 0.67 | |

**Table S7.** Cell Viability EC_50_ (μM). Table reports EC_50_ values calculated for each experiment conducted on cell lines used in the study, after BGA002 treatment, using cell viability assay.

| **Cell Lines** | **MNA** | **Non-MNA** |
| --- | --- | --- |
| NCI-H69 | 4.00 |  |
| NCI-H69 | 3.32 |  |
| NCI-H69 | 2.88 |  |
| NCI-H69 | 3.60 |  |
| H69AR | 5.46 |  |
| H69AR | 4.33 |  |
| H69AR | 4.69 |  |
| NCI-N592 | 3.60 |  |
| NCI-N592 | 3.56 |  |
| NCI-N592 | 4.80 |  |
| NCI-H526 | 4.65 |  |
| NCI-H526 | 3.36 |  |
| NCI-H526 | 6.81 |  |
| GLC-14 | 3.95 |  |
| GLC-14 | 2.00 |  |
| GLC-14 | 2.35 |  |
| DMS 79 |  | 4.52 |
| DMS 79 |  | 5.60 |
| DMS 79 |  | 6.35 |
| Mean | 3.96 | 5.49 |
| Test-T | 0.051 | |
