# Supplementary figures and images for: Antigene MYCN Silencing by BGA002 Inhibits SCLC Progression Blocking mTOR Pathway and Overcomes Multidrug Resistance
Source: Cancers (Basel). 2023 Feb 3;15(3):990. doi: 10.3390/cancers15030990 (PMC9913109; doi:10.3390/cancers15030990)

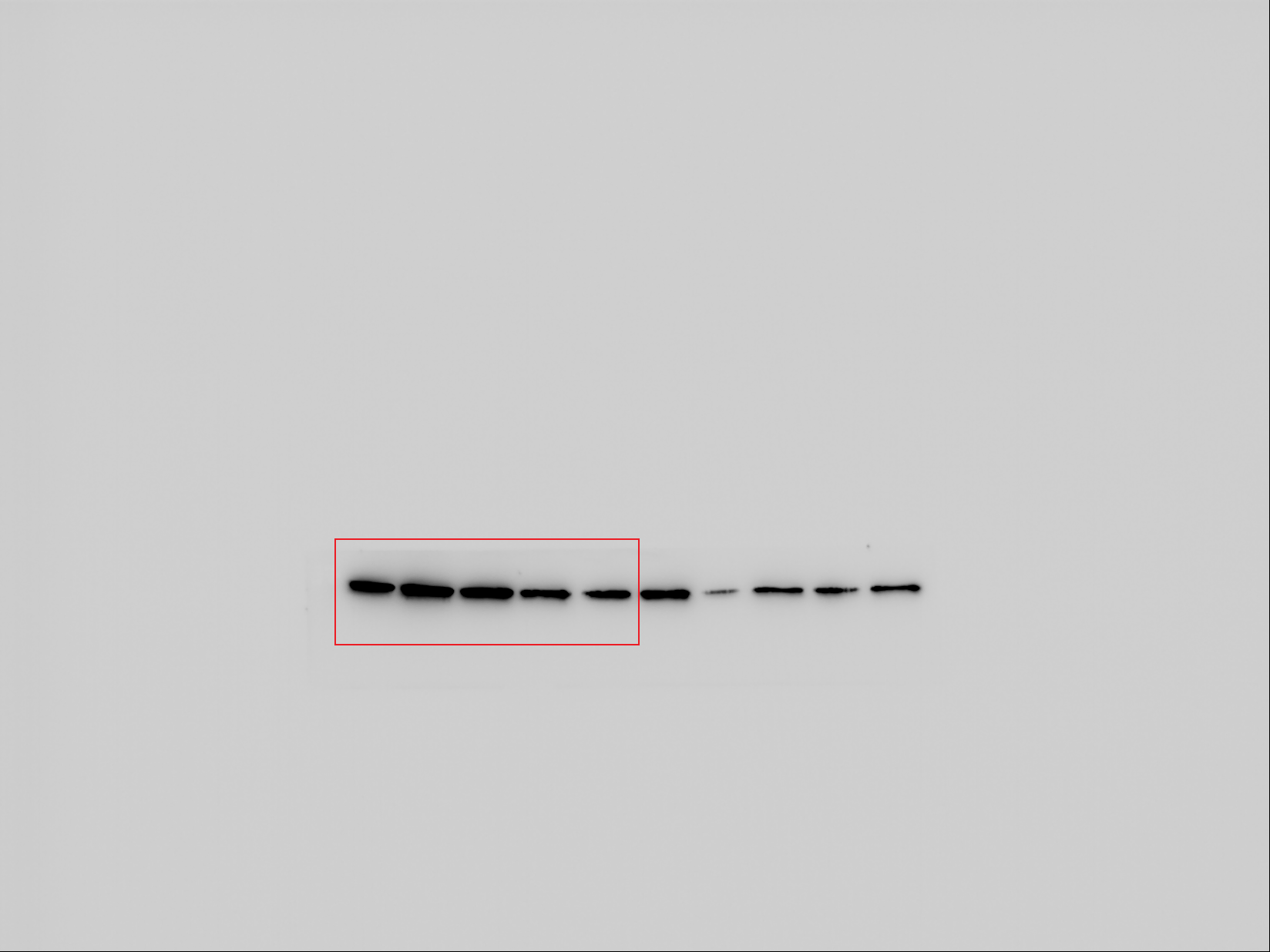

Supplement: Supplementary file 1 [file cancers-15-00990-s001.zip › File S1-Figure 1-C_WB/H69_GAPDH.tiff]

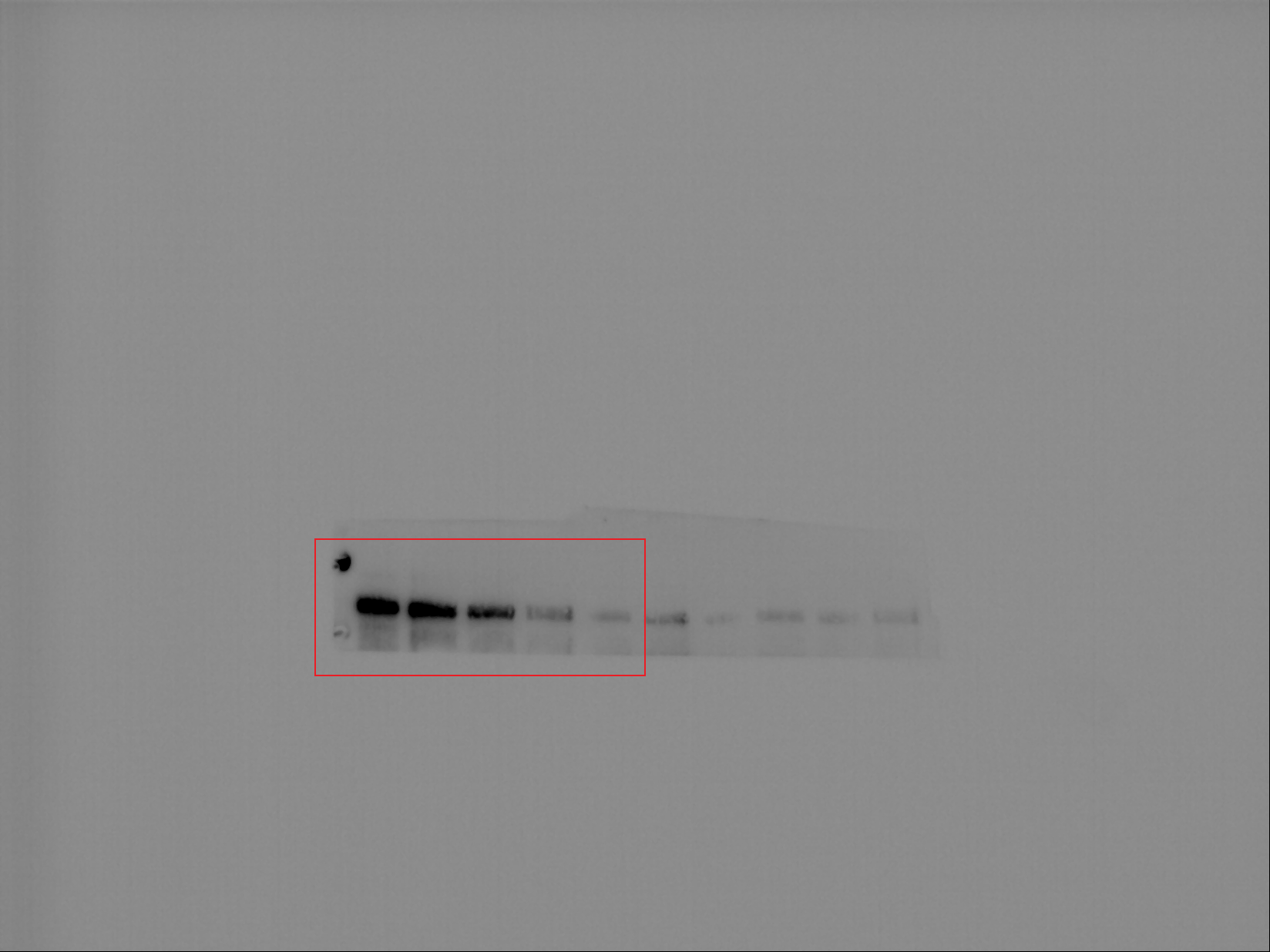

Supplement: Supplementary file 1 [file cancers-15-00990-s001.zip › File S1-Figure 1-C_WB/H69_N-Myc.tiff]

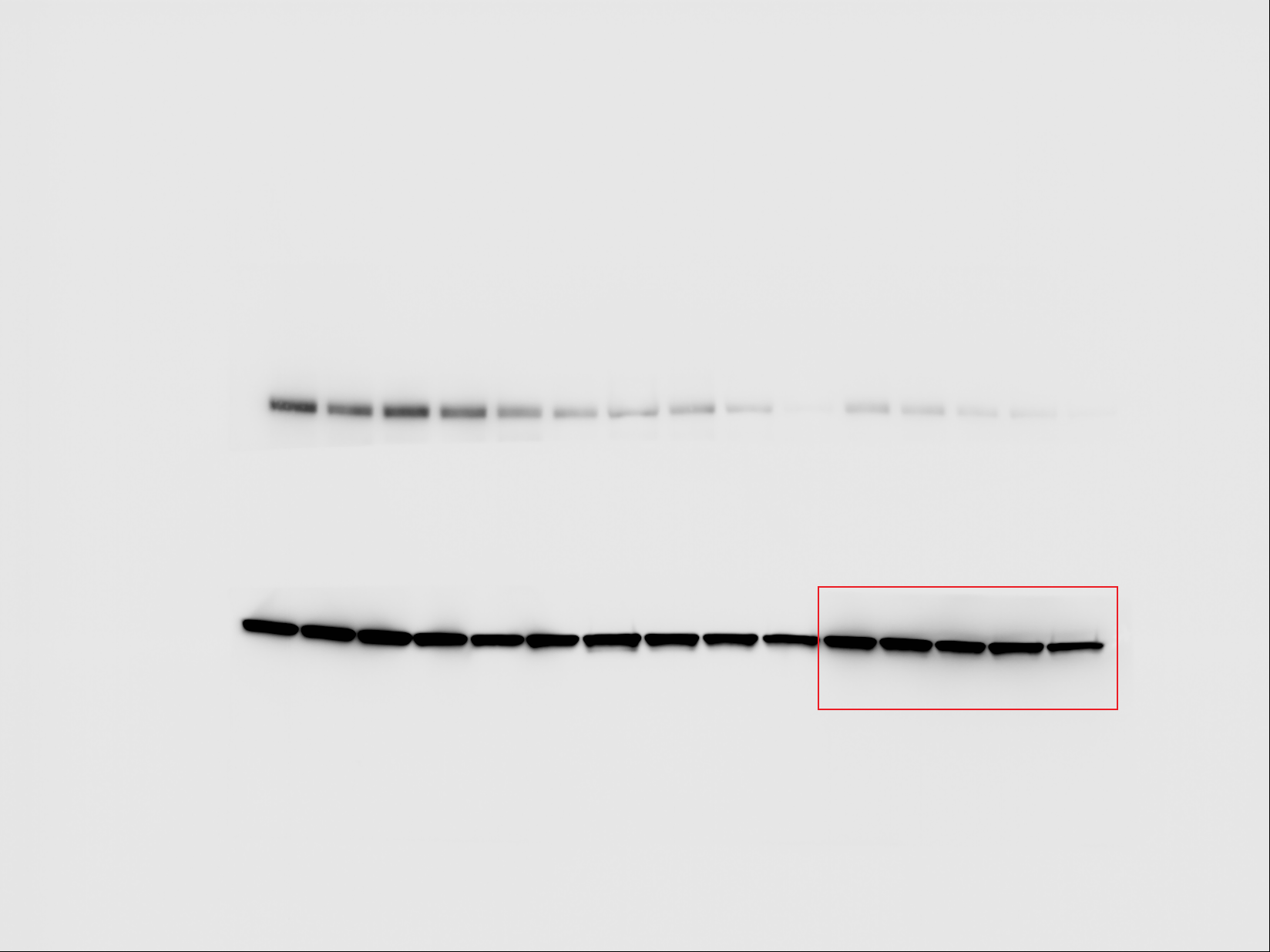

Supplement: Supplementary file 1 [file cancers-15-00990-s001.zip › File S1-Figure 1-C_WB/H69AR_GAPDH.tiff]

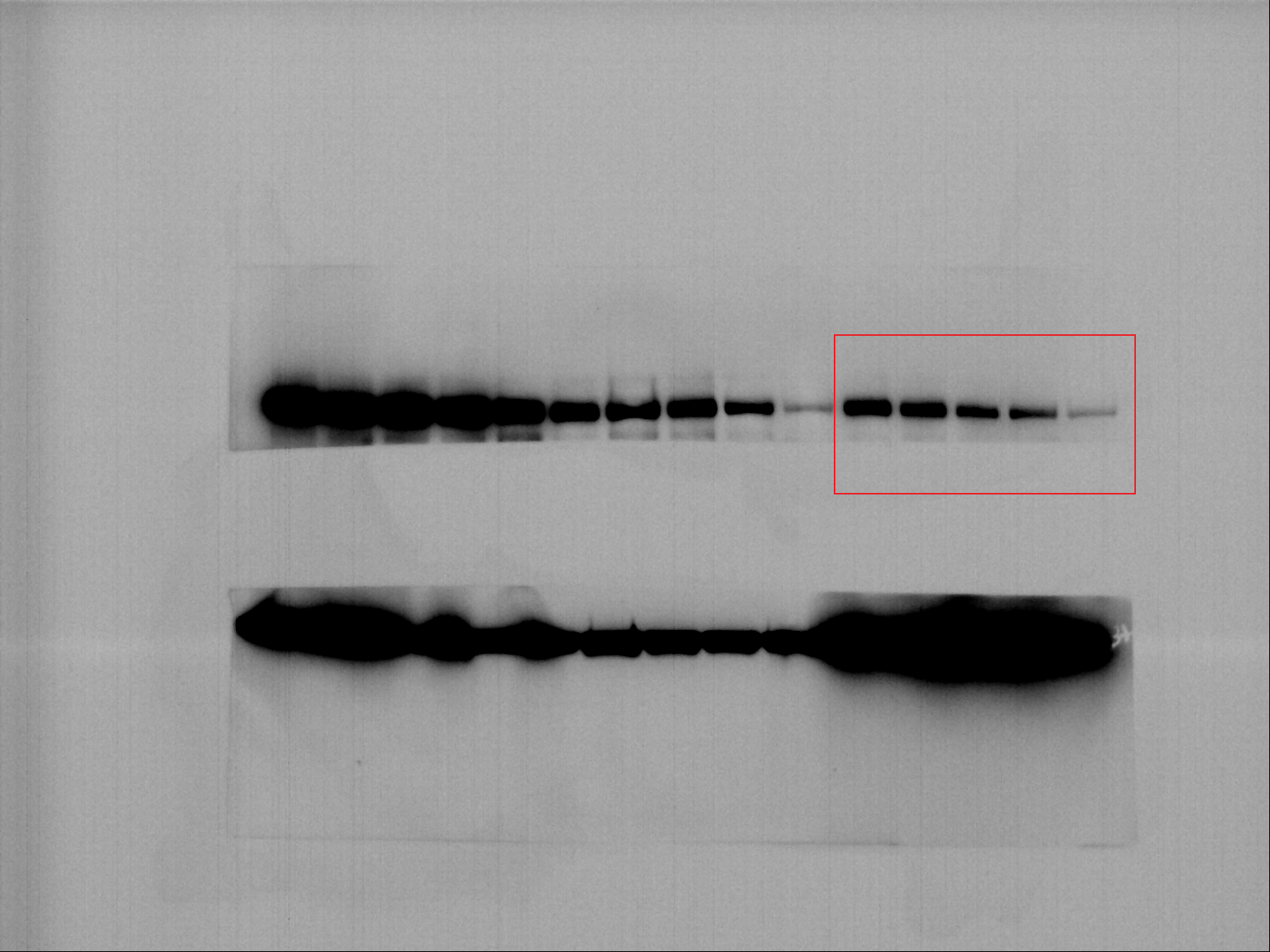

Supplement: Supplementary file 1 [file cancers-15-00990-s001.zip › File S1-Figure 1-C_WB/H69AR_N-Myc.tiff]

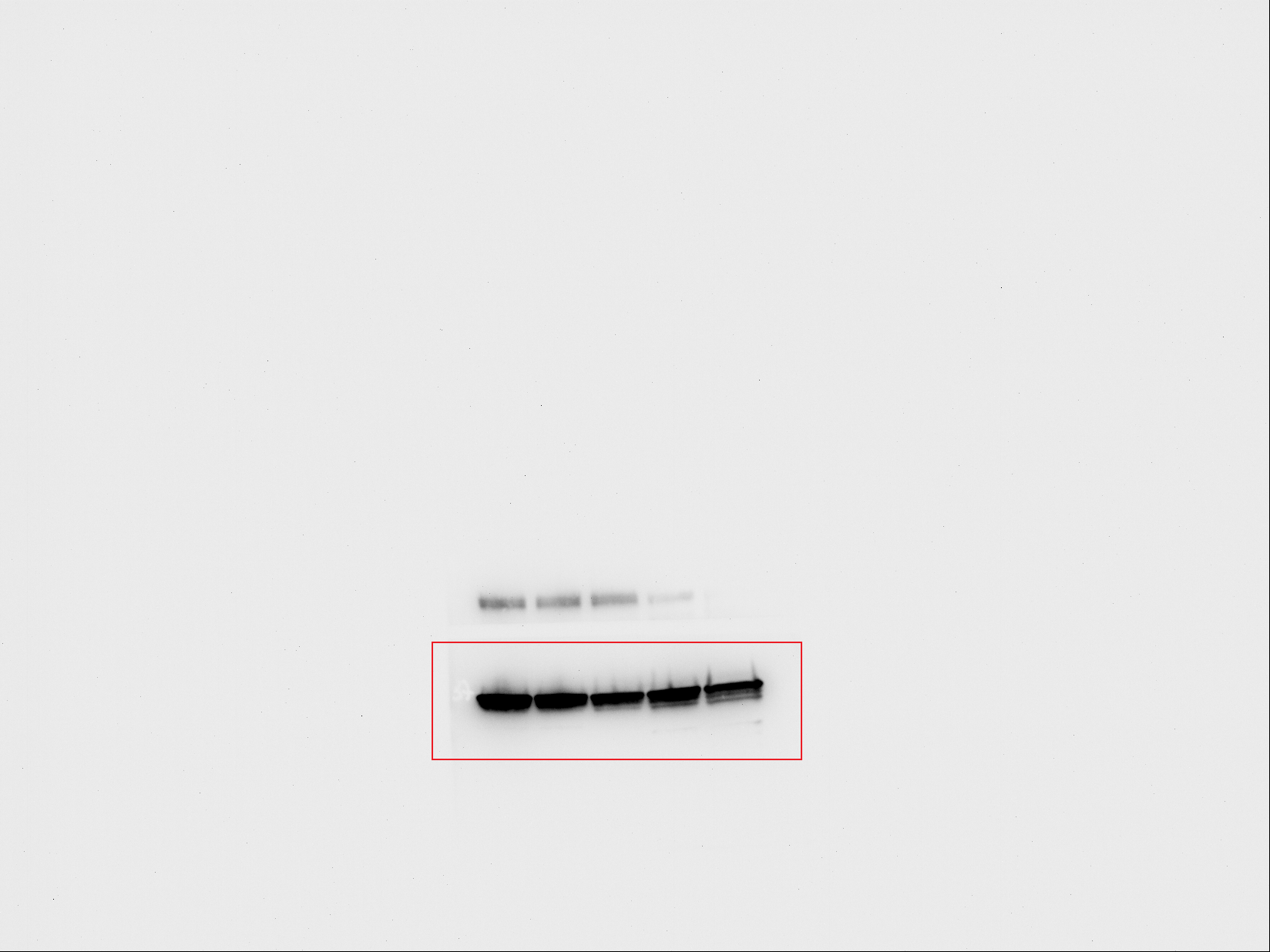

Supplement: Supplementary file 1 [file cancers-15-00990-s001.zip › File S1-Figure 1-C_WB/N592_GAPDH.tiff]

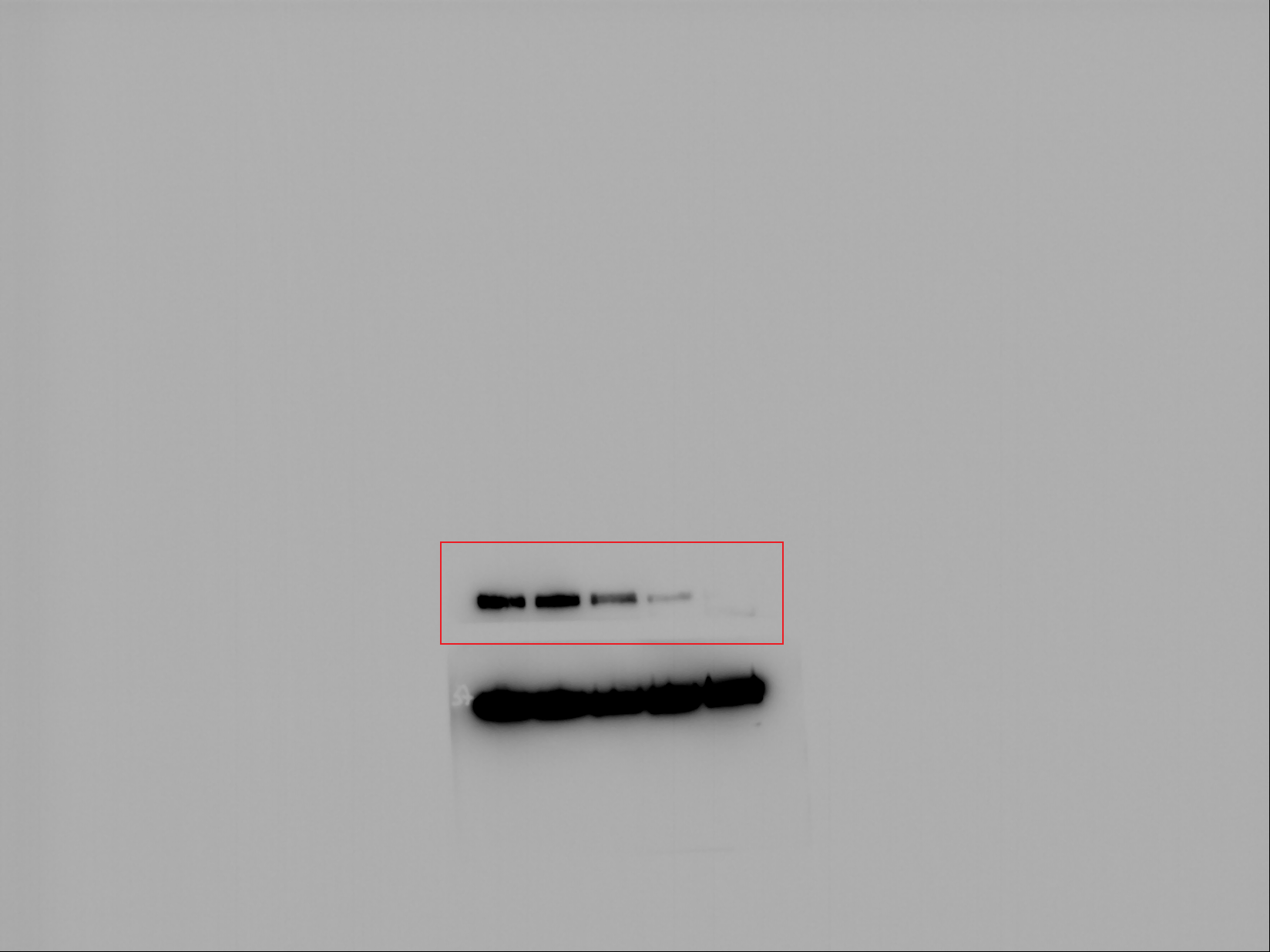

Supplement: Supplementary file 1 [file cancers-15-00990-s001.zip › File S1-Figure 1-C_WB/N592_N-Myc.tiff]

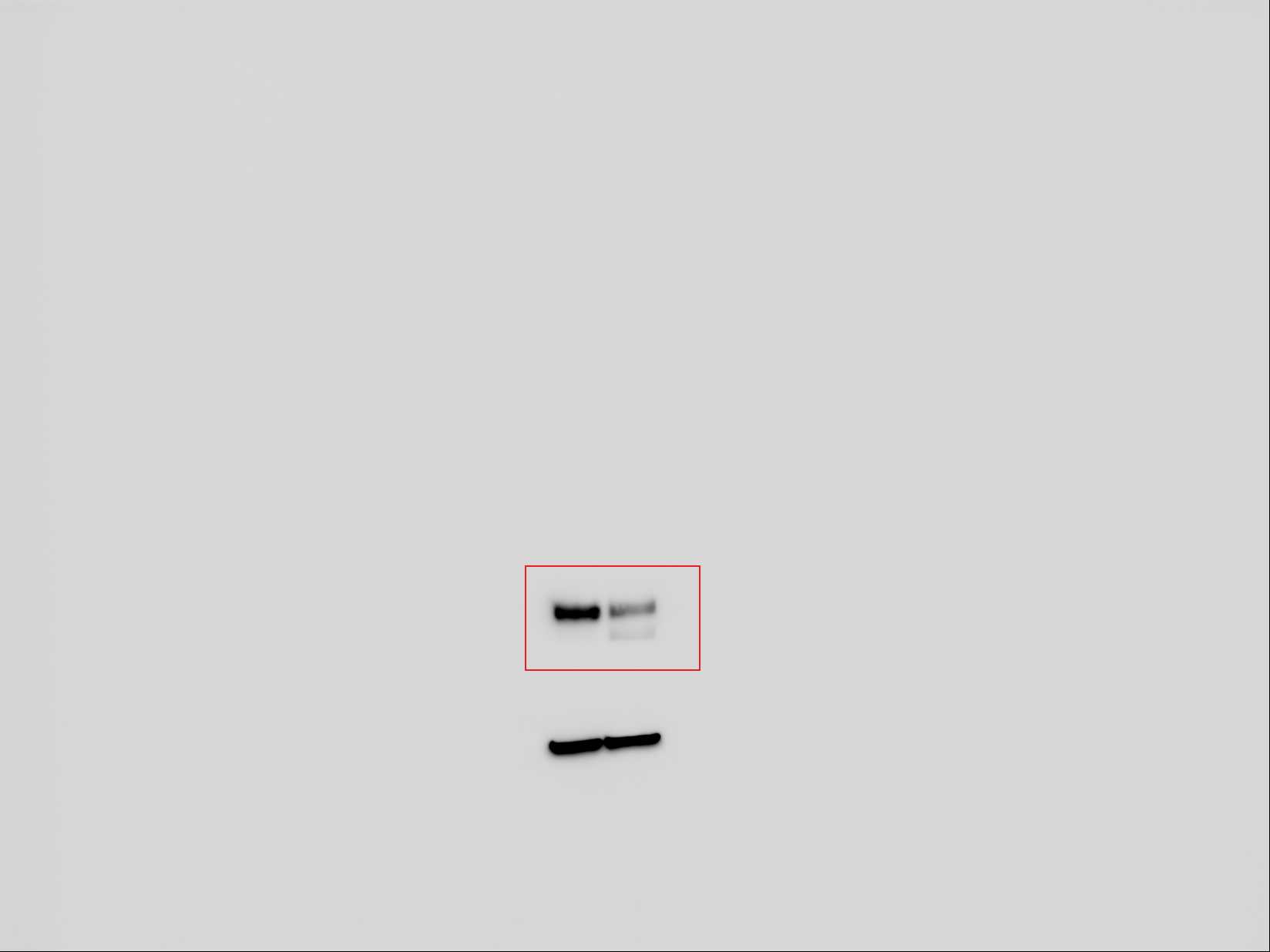

Supplement: Supplementary file 1 [file cancers-15-00990-s001.zip › File S2-Figure 3-C_WB/1.N-Myc.tiff]

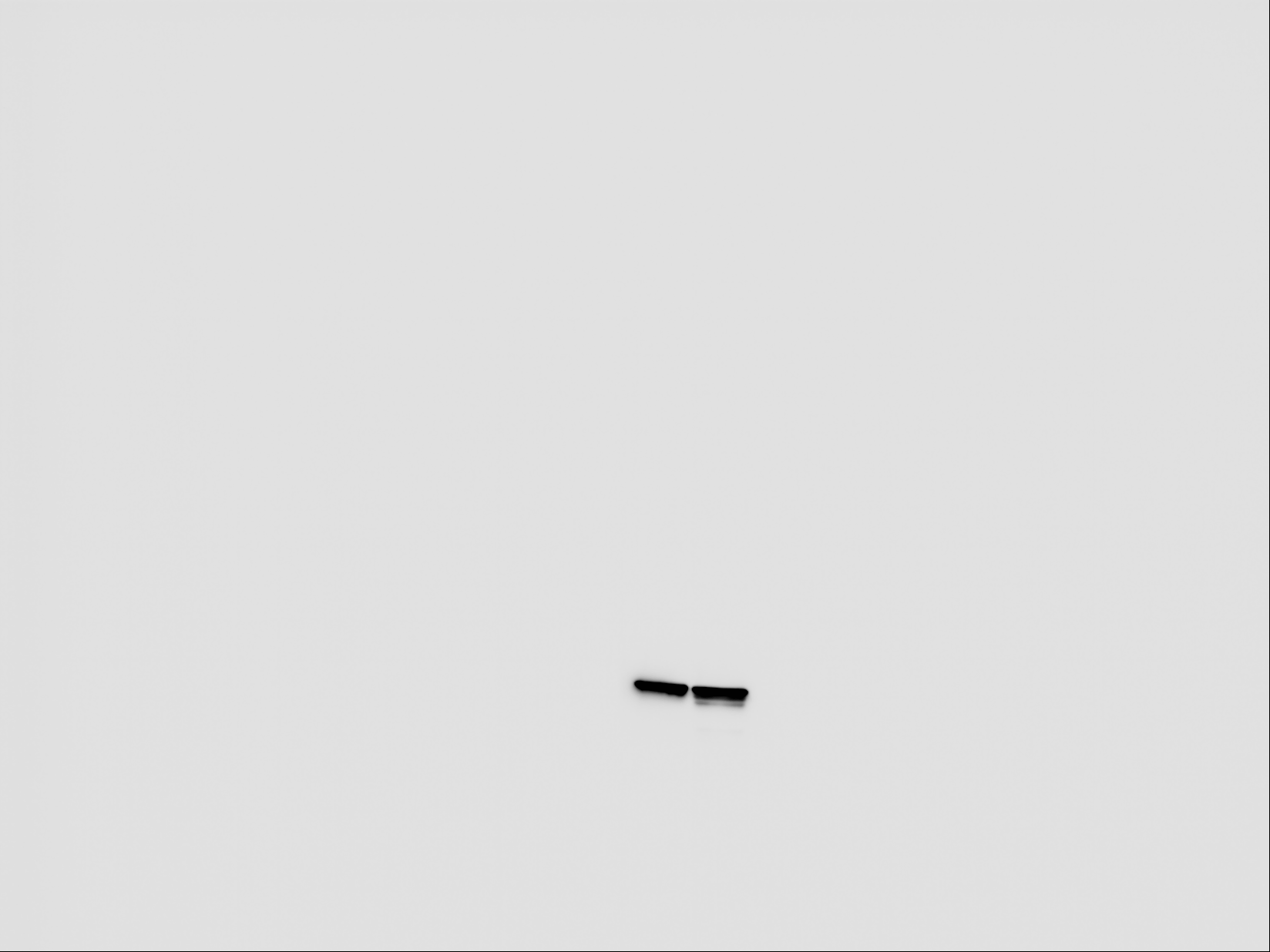

Supplement: Supplementary file 1 [file cancers-15-00990-s001.zip › File S2-Figure 3-C_WB/10.GAPDH.tiff]

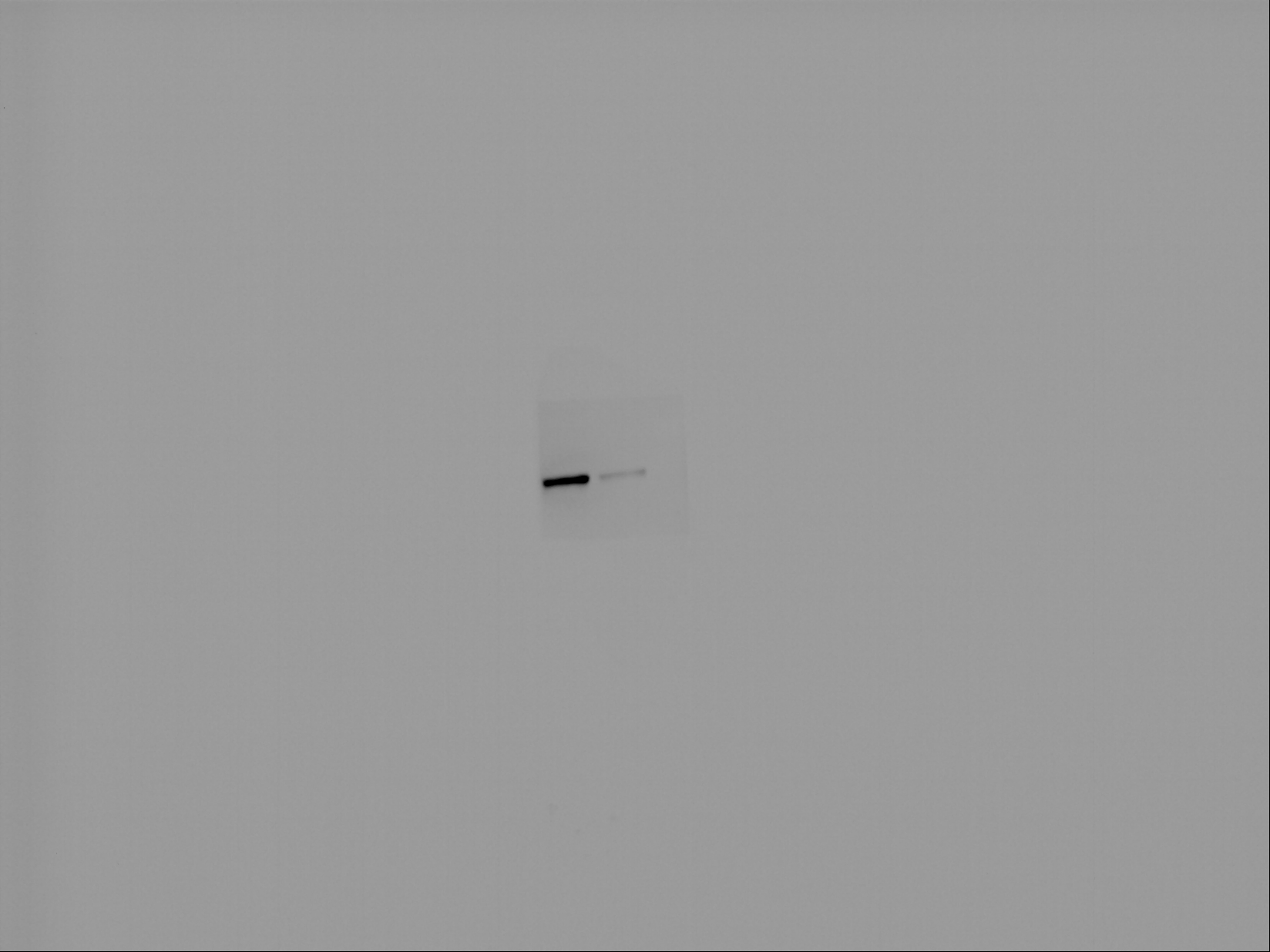

Supplement: Supplementary file 1 [file cancers-15-00990-s001.zip › File S2-Figure 3-C_WB/2.p-AKT_(Ser437).tiff]

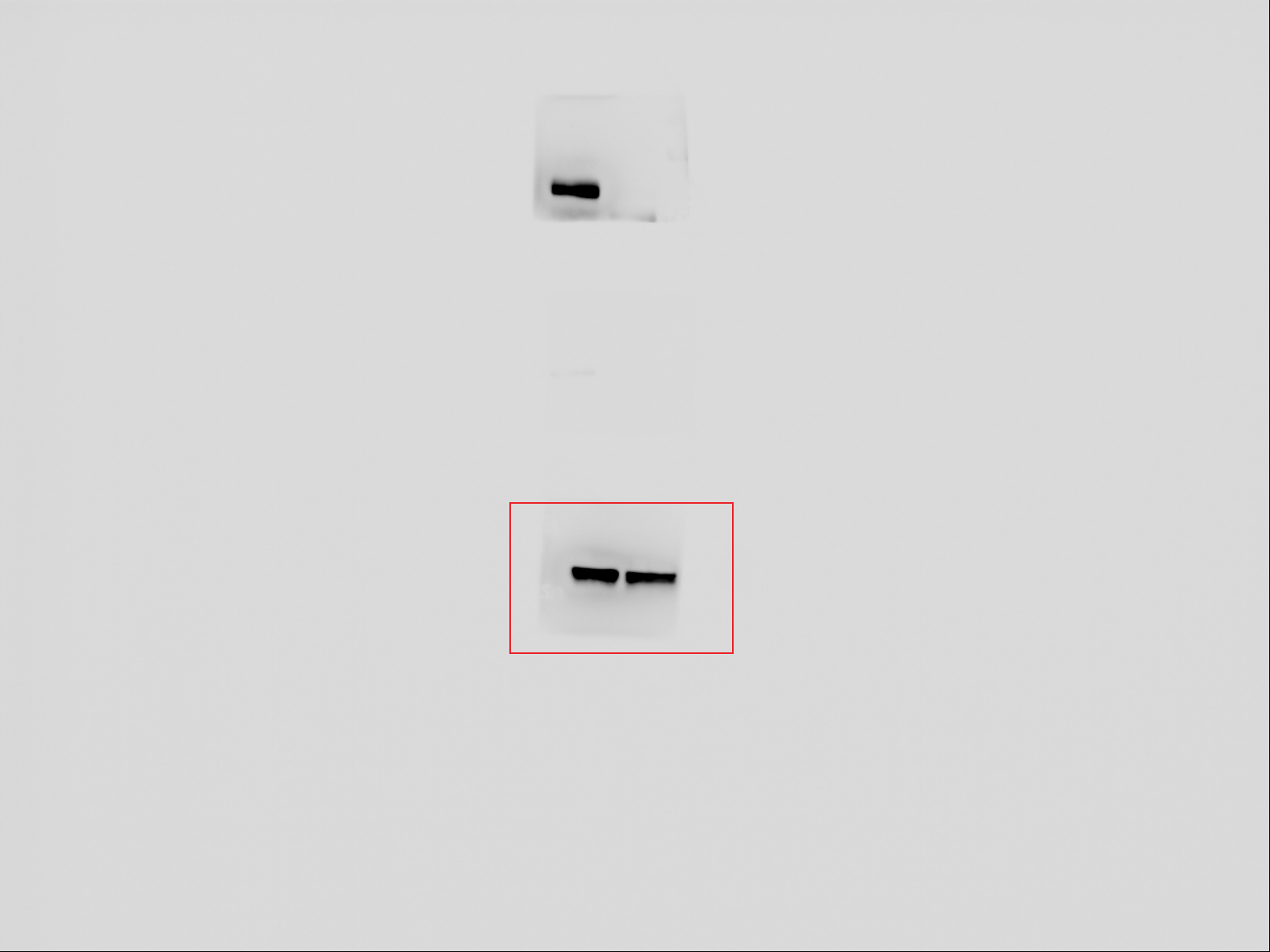

Supplement: Supplementary file 1 [file cancers-15-00990-s001.zip › File S2-Figure 3-C_WB/3.AKT tot .tiff]

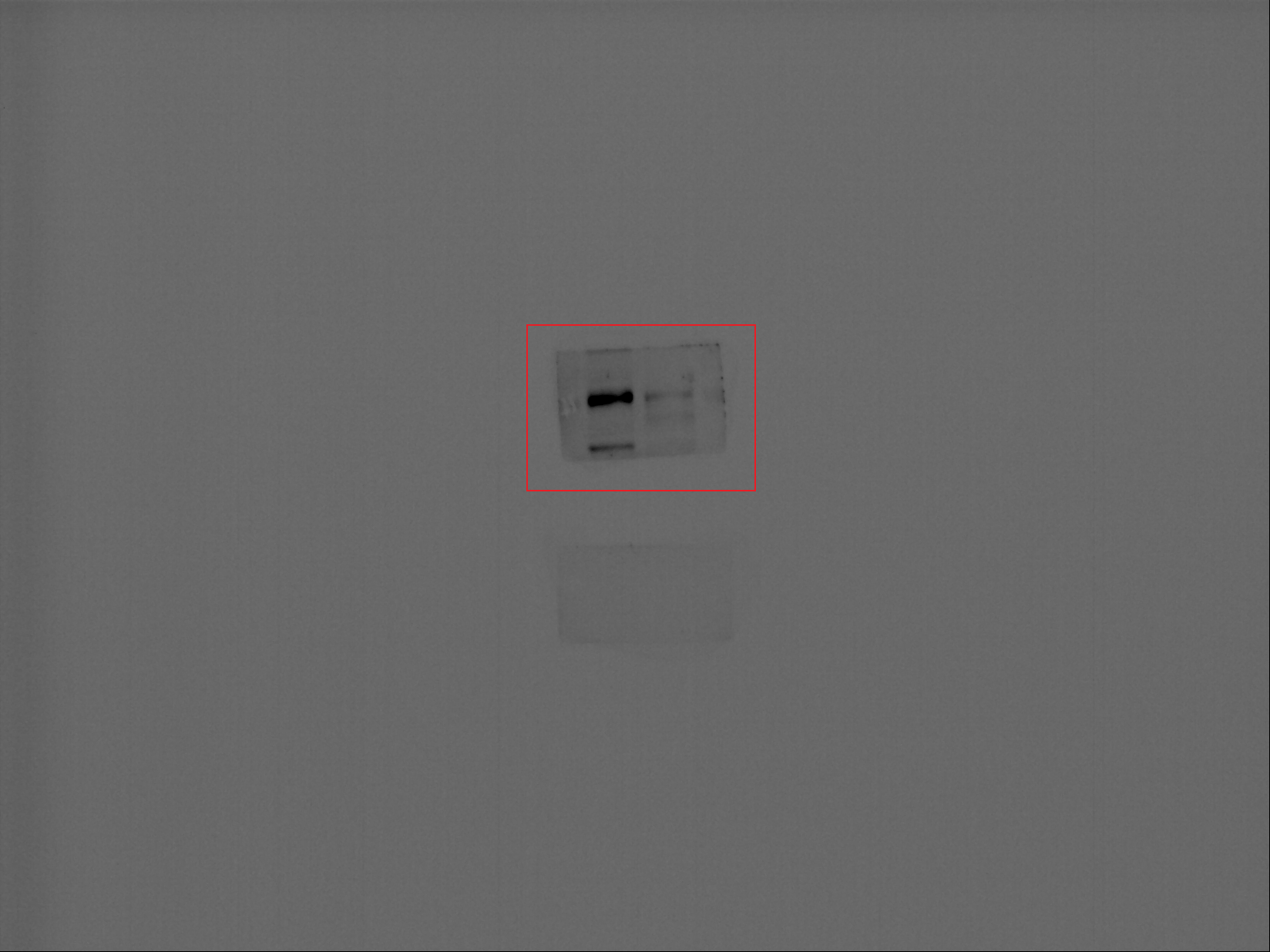

Supplement: Supplementary file 1 [file cancers-15-00990-s001.zip › File S2-Figure 3-C_WB/4.p-P70S6K_(Thr 389).tiff]

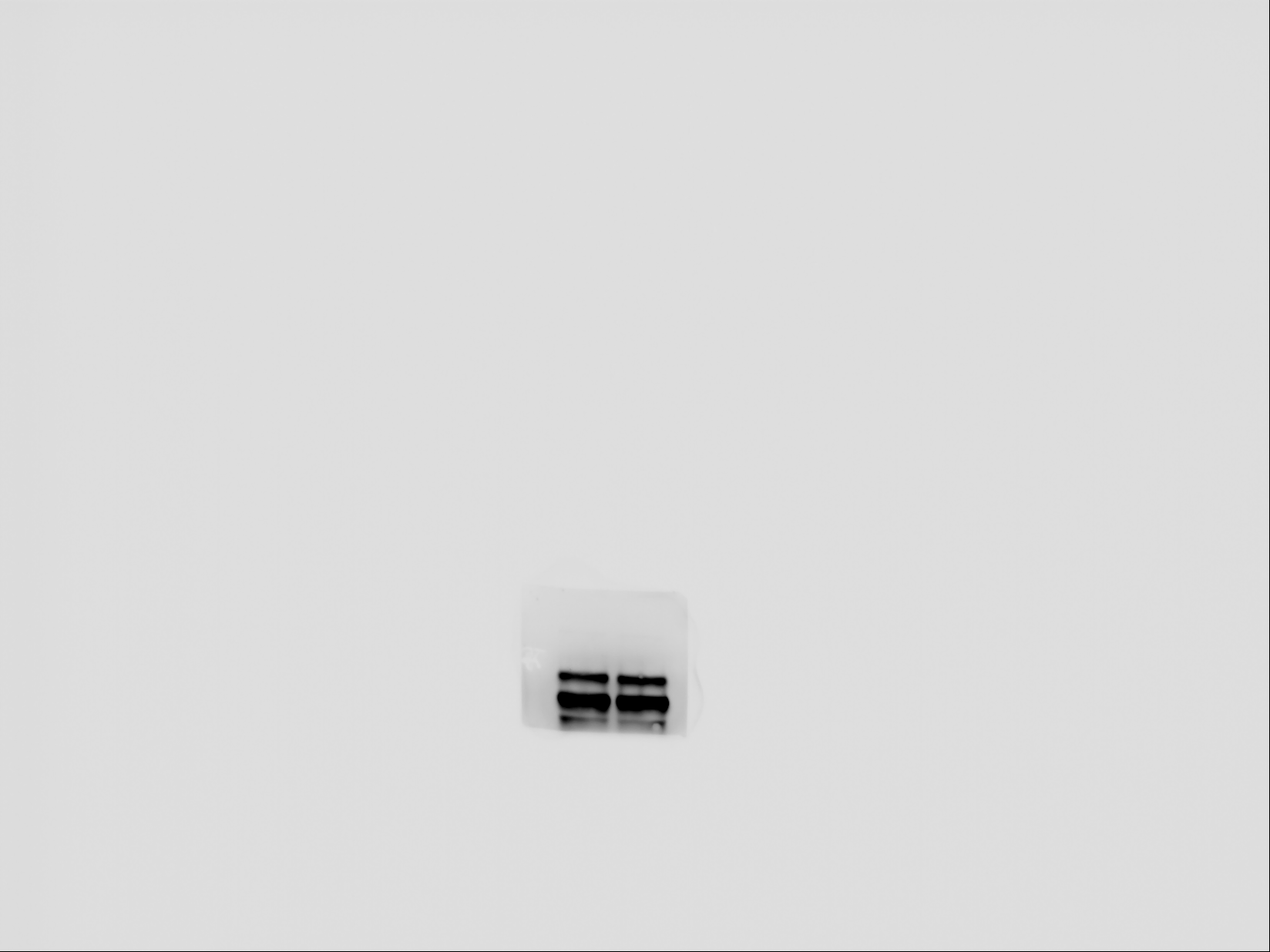

Supplement: Supplementary file 1 [file cancers-15-00990-s001.zip › File S2-Figure 3-C_WB/5.P70S6K tot.tiff]

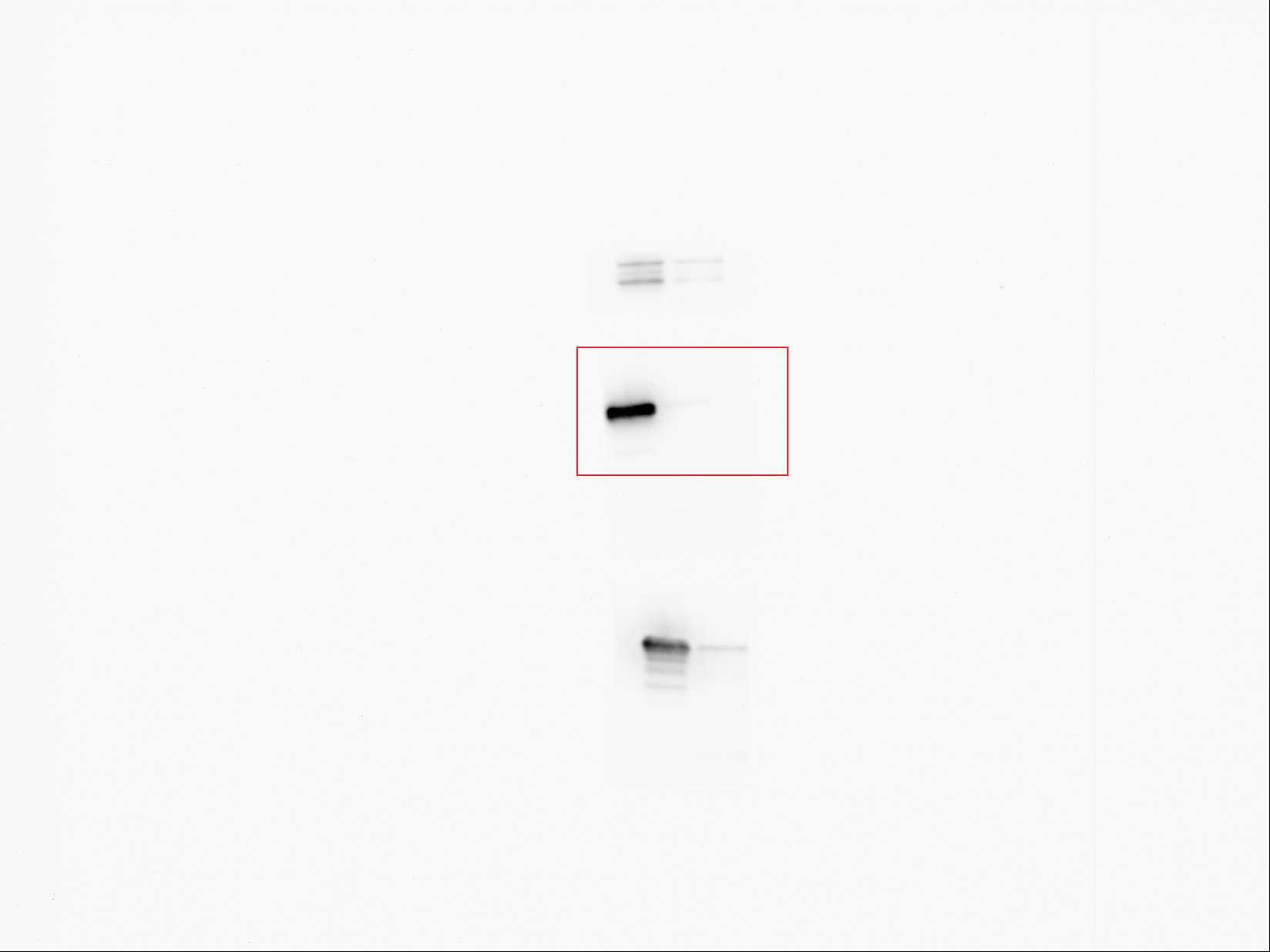

Supplement: Supplementary file 1 [file cancers-15-00990-s001.zip › File S2-Figure 3-C_WB/6.p-S6RP (Ser 235-236).tiff]

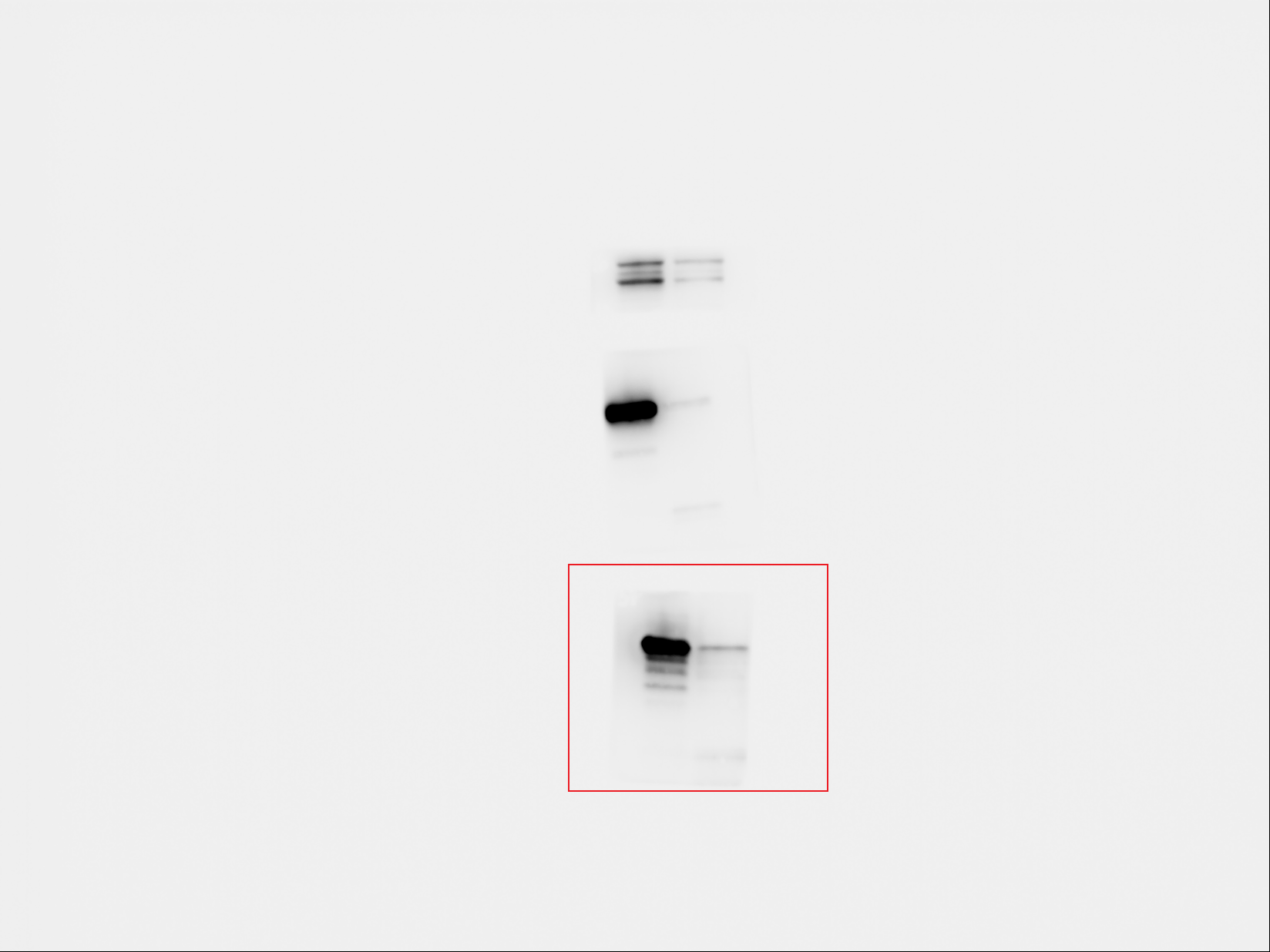

Supplement: Supplementary file 1 [file cancers-15-00990-s001.zip › File S2-Figure 3-C_WB/7.S6RP tot.tiff]

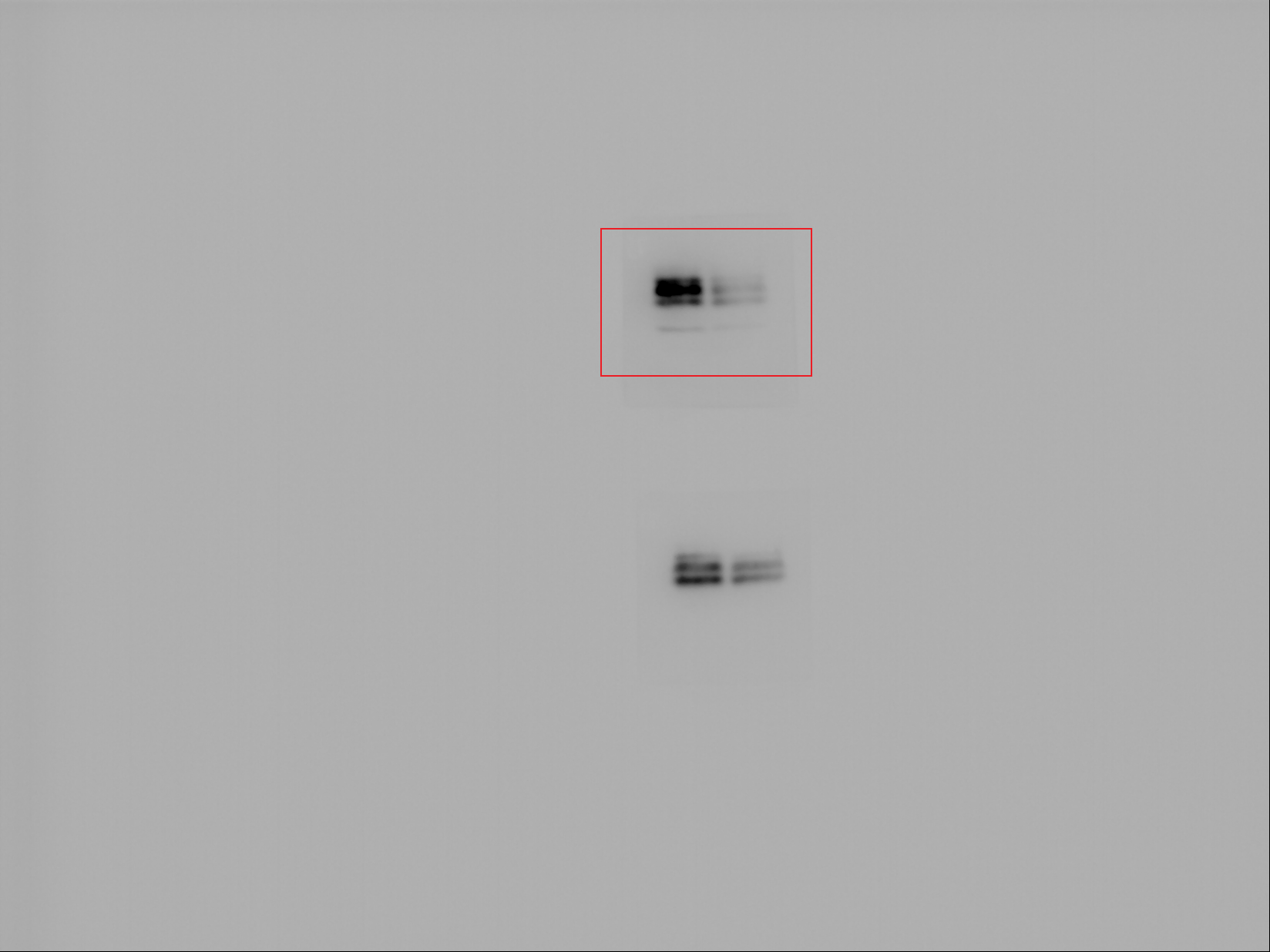

Supplement: Supplementary file 1 [file cancers-15-00990-s001.zip › File S2-Figure 3-C_WB/8.p-4E-BP1_(Thr 37-46).tiff]

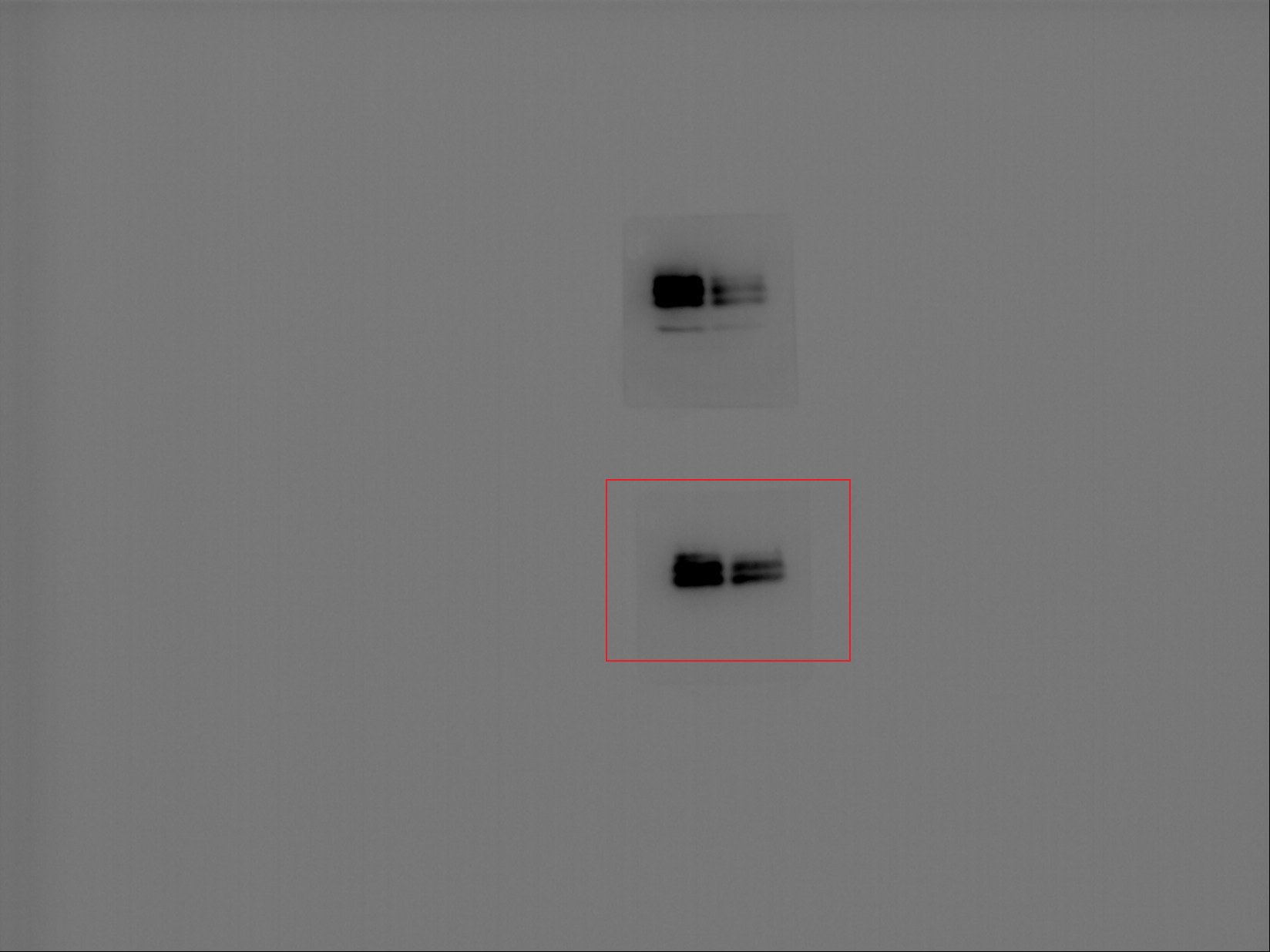

Supplement: Supplementary file 1 [file cancers-15-00990-s001.zip › File S2-Figure 3-C_WB/9.4E-BP1 tot.tiff]
